# A Clinical Consideration of Migraine

**Published:** 1917-02

**Authors:** John A. Litchy

**Affiliations:** Pittsburg, Pa.


### A Clinical Consideration of Migraine.

BY JOHN A. LITCHY, M. PH., M. D., PITTSBURG, PA.

Migraine is considered by the author as the most frequent headache, occurring in 700 of his 15,000 patients sick from all causes. He believes that the so-called acidosis in children may be a forerunner of a well established sick headache habit. The interesting relation between migraine and epilepsy deserve further study. Among the author's 15,000 patients, epilepsy occurred in 7, and both migraine and epilepsy in 70. Auerbach's theory, which attributes migraine to an actual disproportion between skull-capacity and volume of brain, needs further proof. In the *International Clinics* for December, Dr. Litchy shows that the diagnosis is easy when there are headaches which are unilateral, periodical and hereditary, but when only one or two of these symptoms are present, or when there is only a periodicity of some of the minor symptoms or possibly of the aurae, the diagnosis may be difficult. Migraine is frequently mistaken for pelvic disease, for acidosis or cyclical vomiting in children, and organic disease, when some of the aurae are present. The psychasthenic and the gastric symptoms frequently lead to confusion in diagnosis. While the underlying causes of migraine are vague and furnish little light as to treatment, much can be done to ameliorate the symptoms by proper handling of the exciting causes that aggravate the patient's general condition and precipitate the attacks. Most thorough investigation and careful individualization are indicated. Systematic administration of the bromide salts and avoidance of undue fatigue are especially recommended.—*International Clinics.*

